# Comparative evaluation of machine learning and deep learning approaches for compressive strength prediction of geopolymer concrete

**DOI:** 10.1038/s41598-026-50705-w

**Published:** 2026-05-07

**Authors:** H. Ezz, S. M. Bakr

**Affiliations:** 1 Civil Engineering Department, National Research Centre, Giza, Egypt; 2 Al Madinah High Institute for Engineering and Technology, Giza, Egypt

**Keywords:** Machine learning, Deep learning, Artificial neural network, Geopolymer concrete, Compressive strength, Prediction, SHAP, Engineering, Mathematics and computing

## Abstract

Geopolymer concrete has emerged as a promising sustainable alternative to ordinary Portland cement concrete due to its lower environmental impact and favorable engineering performance. However, accurately predicting its compressive strength remains challenging because it is governed by complex interactions among binder composition, activator characteristics, curing conditions, and other mixture parameters. Therefore, reliable data-driven predictive models are needed to support efficient design and performance estimation. For this the study develops a data-driven framework for predicting the compressive strength of geopolymer concrete using both machine learning (ML) and deep learning (DL). A unified database of 594 geopolymer mix designs was compiled from published literature, covering key mix parameters (binder constituents, activator contents, aggregates, water/superplasticizer, fiber contents, and curing age). The dataset was cleaned and divided into 80% training and 20% testing for unbiased evaluation. Multiple supervised ML models (SVM, Decision Tree, Random Forest, Gradient Boosting, XGBoost, LightGBM, and CatBoost) were trained and assessed using MAE, MSE, R^2^, adjusted R^2^, and MAPE, then compared to identify the most reliable predictor. Among ML approaches, CatBoost achieved the best overall performance (MAE = 14.740, MSE = 759.484, R^2^ = 0.968), indicating high accuracy and strong generalization. On the other hand, Decision Tree showed the weakest results among this set, reflected by higher MAE (21.842) and lowest R² (0.912). DL benchmarks were also implemented using ANN Shallow, ANN Deep, and TabNet; the TabNet model emerged as the strongest DL performer, achieving MAE = 18.27, MSE = 765.80, and R^2^ = 0.968, effectively rivaling the best-performing ML approach. Visually, TabNet demonstrated the tightest clustering around the 45° reference line, whereas the ANN Deep model showed the weakest fit and the highest overall error (MAE = 30.15, R^2^ = 0.9106). To enhance interpretability, SHAP was applied to quantify feature contributions and identify the governing variables affecting compressive strength; the results indicate that prediction is dominated by binder and curing related variables, with fibers and minor constituents providing secondary refinements. The study provides a practical predictive tool for strength estimation and supports more efficient geopolymer mix optimization.

**Supplementary Information:**

The online version contains supplementary material available at 10.1038/s41598-026-50705-w.

## Introduction

Geopolymer concrete (GPC) has gained increasing attention as a sustainable alternative to Portland Cement Concrete (PCC), whose production is energy-intensive and contributes approximately 5–7% of global anthropogenic CO₂ emissions^1–4^. GPC is synthesized through geopolymerization, a chemical process in which aluminosilicate-rich precursors such as fly ash and metakaolin are activated using alkaline solutions, typically sodium hydroxide and sodium silicate, to form a hardened binder^5^. This concept, introduced by Joseph Davidovits in 1978, forms the foundation of geopolymer science^6^. During this process, dissolved silicate and aluminate species undergo polycondensation to form a three-dimensional Si–O–Al–O network, resulting in sodium alumino-silicate hydrate (N-A-S-H) gels that provide high early strength and reduced shrinkage compared with traditional C-S-H binders. GPC can therefore achieve compressive strengths equal to or greater than those of conventional Portland cement concrete, while offering reduced environmental impact and potentially lowering CO₂ emissions by up to 70%^3,4^. Despite these advantages, GPC is inherently brittle and exhibits relatively low tensile capacity, limited fracture toughness, and reduced impact resistance, which restrict its structural application^7^. Like other quasi-brittle materials, geopolymer concrete may develop microcracks and fail without significant plastic deformation when subjected to overload, particularly under tensile or impact-related stresses^4^. To address this limitation, researchers have explored fiber-reinforced geopolymer concrete (FRGC) by incorporating discrete fibers into the matrix. Steel fibers can enhance flexural strength, impact resistance, toughness, and post-cracking load-carrying capacity by bridging cracks, while polypropylene (PP) fibers improve crack resistance and contribute to a less brittle failure mode. Accordingly, the incorporation of steel and polypropylene fibers can improve the suitability of geopolymer concrete for structural applications^8^. However, predicting the compressive strength of geopolymer concrete remains a complex task because of the nonlinear interactions among multiple variables, including binder composition, alkali activator concentration, curing conditions, aggregate content, water content, and fiber reinforcement. Traditional empirical models are often inadequate for capturing these intricate relationships. In contrast, machine learning (ML) algorithms such as Random Forest, Support Vector Machines (SVM), Decision Trees, and ensemble methods have shown strong capability in modeling nonlinear systems with high accuracy^9–15^. While several studies have applied ML techniques to predict the performance of GPC, few have examined mixtures reinforced simultaneously with both steel and polypropylene fibers. Furthermore, the influence of key mix parameters such as fiber type and dosage, sodium hydroxide molarity, and curing age on compressive strength remains under-investigated in fiber-hybrid systems. Understanding these interactions is essential for developing accurate predictive models and optimized concrete formulations. Deep learning (DL) extends ML by using multi-layer neural networks to learn complex nonlinear input–output relationships directly from data. These models are typically trained through backpropagation using gradient-based optimizers such as Adam, while regularization strategies such as dropout are employed to reduce overfitting and improve generalization^16^. In cementitious and geopolymer systems, DL models are particularly relevant because compressive strength depends on strongly coupled nonlinear interactions among precursor contents, activator dosage and concentration, water content, aggregates, fibers, and curing age. Previous studies have successfully applied ANN-based and other data-driven approaches to predict the compressive strength of fly ash- and geopolymer-based concretes with high accuracy, thereby reducing trial-and-error experimentation and accelerating mix-design screening^9^. Beyond conventional feed-forward ANNs, modern DL architectures designed for tabular datasets, such as TabNet, employ sequential attention mechanisms that identify influential features at each decision step. This can provide competitive predictive performance while also improving interpretability, making such models attractive for concrete mix-design optimization studies.

Recent research in data-driven construction materials has moved beyond conventional ensemble models toward hybrid and explainable frameworks that improve both prediction accuracy and engineering interpretability. Recent studies have explored optimized ML pipelines, chemically informed feature sets, and attention-based tabular learning to predict compressive strength more efficiently in sustainable concrete systems. In parallel, explainable AI (XAI) is increasingly being used to support transparent mix design by identifying governing variables and quantifying their nonlinear effects. Beyond SHAP, tools such as permutation importance, partial dependence plots, accumulated local effects, and local surrogate explanations are being used to improve confidence in model-driven material optimization. These developments motivate the present work, which combines comparative ML/DL evaluation with post hoc interpretability for geopolymer concrete strength prediction^17^.

Recent studies have also highlighted the growing importance of explainable data-driven frameworks in structural and materials engineering. For example, Abbood et al. applied enhanced CatBoost with SHAP and partial dependence analysis to shear-strength prediction in reinforced concrete deep beams, while a subsequent study by the same authors developed an explainable boosting-based ensemble framework coupled with Bayesian optimization, further emphasizing the value of interpretable and optimized ML models in civil engineering prediction tasks^18,19^.

Also studies have further expanded the role of AI-based and sustainability-oriented frameworks in concrete research, including machine-learning prediction of recycled concrete powder systems, ANN-based optimization of concrete strength properties, prediction of structural concrete behavior, and environmental assessment of waste-based concrete materials, thereby reflecting the growing integration of predictive modeling and sustainability evaluation in modern concrete engineering^20–22^.

As shown in Table 1, previous studies cited in this manuscript confirm the potential of machine-learning methods for predicting compressive strength in geopolymer and related cementitious materials. However, most prior studies focused on either limited algorithm sets, specific material systems, or conventional ANN-based modeling. In contrast, the present study provides a broader comparative framework integrating both machine learning and deep learning models with SHAP-based interpretation on a unified geopolymer concrete dataset.

**Table 1:** Comparison of previous studies and the present study on geopolymer concrete strength prediction.

The study	Material/study focus	Algorithms/models used	Study type	Main relevance to present study	Difference from present study
Ahmad et al. (2021)^9^	Geopolymer concrete compressive-strength prediction	Novel machine-learning algorithms	Direct GPC prediction study	Demonstrates that ML can effectively predict GPC compressive strength	Present study uses a larger unified literature-based dataset, compares more ML models, adds DL models, and includes SHAP interpretation.
Emarah (2022)^13^	Fly ash-based geopolymer concrete compressive-strength analysis	Machine-learning approaches	Direct GPC prediction study	Supports the use of data-driven methods for geopolymer strength estimation	Present study expands beyond fly ash-based systems and compares ML and DL models under one framework.
Chaabene et al. (2020)^11^	Mechanical properties of concrete	Machine learning	Review/background study	Provides general evidence that ML is suitable for predicting concrete properties	Present study is more specific, focusing on geopolymer concrete compressive strength rather than concrete properties in general.
Ahmad et al. (2021)^10^	Concrete with supplementary cementitious materials	Advanced ML approaches	Related concrete prediction study	Supports the broader applicability of ML in sustainable cementitious materials	Present study specifically targets geopolymer concrete and includes DL benchmarks in addition to ML.
Ahmad et al. (2021)^12^	Recycled aggregate concrete compressive-strength prediction	GEP and ANN	Related concrete prediction study	Supports ANN-based prediction of compressive strength in cementitious materials	Present study focuses on geopolymer concrete and uses a broader ML/DL comparison framework.
Song et al. (2021)^14^	Ceramic waste-based concrete compressive strength	ANN	Related concrete prediction study	Provides ANN-based evidence for compressive-strength prediction in alternative concrete systems	Present study extends beyond ANN to boosting models and TabNet for geopolymer concrete.
Song et al. (2021)^15^	Fly ash-admixed concrete compressive strength	Machine-learning algorithms	Related concrete prediction study	Reinforces the effectiveness of ML for fly ash-containing sustainable concrete materials	Present study is centered on geopolymer concrete and includes explainable AI through SHAP.
Sathiparan et al. (2025)^17^	Pervious concrete properties	Machine-learning review	Review/background study	Supports the recent trend toward data-driven materials prediction and interpretability	Present study is a primary research study focused on geopolymer concrete compressive strength with direct model development and testing.
Present study	Geopolymer concrete compressive-strength prediction using a literature-based dataset of 594 mixes	SVM, DT, RF, GB, XGBoost, LightGBM, CatBoost, ANN-Shallow, ANN-Deep, TabNet	Direct GPC prediction study	Provides a broad comparative ML/DL framework with SHAP-based interpretation	Integrates conventional ML, deep learning, and explainable AI in one unified workflow.

Recent studies have further demonstrated the value of computational modeling for geopolymer concrete strength prediction and mixture optimization. Shahmansouri et al. developed an artificial neural network model to predict the compressive strength of eco-friendly geopolymer concrete incorporating silica fume and natural zeolite, showing the effectiveness of ANN-based modeling for sustainable geopolymer systems. In another study, gene expression programming was used to predict the compressive strength of eco-efficient GGBS-based geopolymer concrete, providing an interpretable symbolic-regression framework for strength estimation. Response surface methodology has also been applied to optimize the mechanical performance of GGBFS-based geopolymer concrete containing natural zeolite and silica fume, highlighting the importance of statistical optimization in mixture design. These studies confirm the growing role of data-driven and computational approaches in geopolymer concrete research. Building on this progress, the present study provides a broader comparative assessment of multiple ML and DL models on a unified literature-based dataset and further enhances model transparency through SHAP-based interpretation^23–25^.

The primary objective of this study is to develop and assess robust machine learning (ML) and deep learning (DL) models for predicting the compressive strength of geopolymer concrete using a comprehensive literature-based database. A total of 594 geopolymer concrete mix designs were compiled from published studies and organized into a unified dataset containing the most consistently reported mix parameters (binder^17^ constituents, activator contents, aggregates, water, superplasticizer, fibers, and curing age). Several supervised ML algorithms SVM, Decision Tree, Random Forest, Gradient Boosting, XGBoost, LightGBM, and CatBoost were implemented and evaluated. In addition, DL benchmarks were developed using ANN Shallow, ANN Deep, and TabNet architectures. Model performance was quantified using R², adjusted R², MSE, MAE, and MAPE, and the models were compared to identify the most accurate and reliable predictor. To enhance interpretability and extract engineering insight, SHAP (Shapley Additive Explanations) was applied to quantify the contribution of each input variable and rank the governing parameters influencing compressive strength. The outcome of this work is a practical, data-driven framework that supports reliable strength estimation and aids in future geopolymer concrete mix optimization. Therefore, the objectives of this study can be summarized as:

- To compile a unified literature-based dataset (594 mixes) of geopolymer concrete mix designs suitable for data-driven strength prediction.
- To develop and compare multiple supervised ML models (SVM, Decision Tree, Random Forest, Gradient Boosting, XGBoost, LightGBM, CatBoost) and DL models (ANN Shallow, ANN Deep, TabNet) for compressive strength prediction.
- To evaluate and rank model performance using statistical indicators (R², adjusted R², MSE, MAE, and MAPE) and identify the most reliable model.
- To interpret model predictions using SHAP to quantify feature contributions and determine the most influential mix parameters controlling compressive strength.
- To establish a practical predictive framework that reduces trial-and-error in geopolymer mix design and supports more efficient and sustainable mixture optimization.

## Materials and methods

**Fig. 1:**
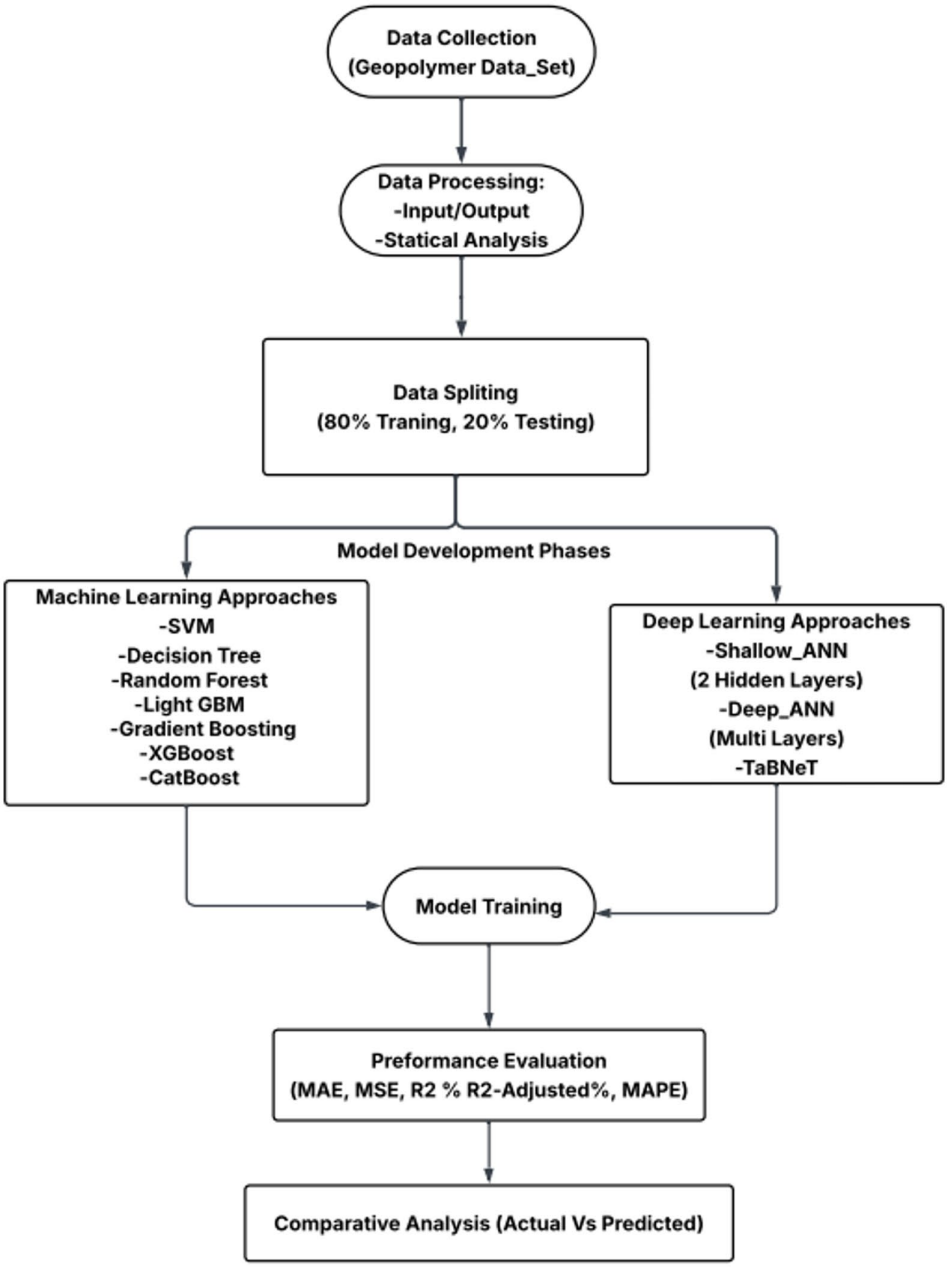
Geopolymer strength prediction workflow.

This study applies a data driven framework to predict the compressive strength of geopolymer concrete using machine learning and deep learning. A dataset of 594 mix designs was compiled from published literature, cleaned, and split into 80% training and 20% testing for unbiased evaluation. Multiple ML models (SVM, Decision Tree, Random Forest, Gradient Boosting, XGBoost, LightGBM, and CatBoost) and two ANN architectures (shallow and deep) were developed and assessed using MAE, MSE, R², adjusted R², and MAPE. The best-performing models were identified based on combined error and goodness of fit measures. Model interpretability was addressed using SHAP to quantify feature contributions and rank the most influential variables. The overall workflow is summarized in Fig. 1.

### Material properties

The dataset used in this study was compiled exclusively from published literature and includes 594 geopolymer concrete mix designs. The database covers mixtures produced using common geopolymer precursors such as fly ash and metakaolin, with additional binder components frequently reported in the literature, including slag and silica fume. The alkaline activation system is represented through variables describing the activator constituents and concentration, including NaOH dosage, sodium silicate (Na₂SiO₃) content, and alkaline molarity. In addition, the dataset includes mixture constituents that directly influence fresh and hardened behavior, such as fine and coarse aggregate contents, water content, and superplasticizer dosage. To account for the influence of reinforcement on strength development, the database also incorporates polypropylene fiber (PP) and steel fiber (SF) contents as input parameters. The curing condition is represented by age (days), while the output variable is the compressive strength of geopolymer concrete. A summary of the variables, units, and value ranges used in this study is provided in Table 3, and the corresponding material chemical compositions (as reported in the collected sources) are summarized in Table 2.

**Table 2:** Chemical analysis of binder types.

%Compound	Meta-kaolin	Fly Ash	Slag	Silica Fume
SiO_2_	57.16	64.71	34.86	96.00
TiO_2_	2	1.43	1.91	0.08
Al_2_O_3_	26.15	25.42	13.93	0.72
Fe_2_O_3_	4.79	4.19	0.20	0.1
MnO	0.02	0.03	1.45	0.09
CaO	4.44	1.12	36.59	0.27
Na_2_O	0.03	0.02	-	0.26
K_2_O	0.47	0.89	1.01	0.38
P_2_O_5_	0.07	0.24	-	0.1
Cl	0.21	0.04	-	0.1
SO_3_	0.65	0.23	2.70	0.19
MgO	0.26	0.27	6.04	0.4
Loss of ignition (LOI)	3.44	0.33	1.19	1.00

### Dataset collection

A consolidated dataset of 594 geopolymer concrete mix designs was compiled from published studies to develop the machine learning and deep learning models for compressive strength prediction. a total of 594 concrete mix designs were collected from published research. The data set collected were obtained from 19 previously published research sources^4,8,17–32^. The dataset comprises the most frequently reported geopolymer concrete mix-design parameters and curing age, which were used as model inputs, while compressive strength was adopted as the output variable. The collected mixes cover a wide range of binder compositions, including fly ash, slag, metakaolin, and silica fume, in addition to alkaline activators (NaOH and Na₂SiO₃), aggregates (gravel and sand), water, superplasticizer, and fiber contents (PP and SF). As reflected by the descriptive statistics in Table 2, the variables exhibit substantial variability across the compiled studies, confirming that the database represents diverse mix designs and material proportions rather than a narrow experimental program. To support robust ML/DL training while maintaining consistency across sources, the dataset was restricted to the most commonly available parameters reported in the literature, which reduces missing data and limits unnecessary diversity in model inputs. Table 2 summarizes the full statistical description of each variable (mean, standard deviation, minimum, median, and maximum), providing a clear overview of the input ranges and the corresponding spread of compressive strength values used for model development and evaluation.

To improve dataset reliability, the compiled records were also checked for repeated entries that could arise from duplicated reporting of identical mix designs across literature sources. Where clear duplicates were identified, only one representative record was retained. The dataset was further reviewed for anomalous values by checking reported ranges, units, and consistency with the original source data. No automatic outlier deletion was performed unless a record was clearly incomplete or inconsistent with the source information, in order to preserve the natural variability of the literature-based dataset.

After variable harmonization, the compiled records were screened for completeness. Only samples containing all selected input variables and the target compressive strength were retained in the final database. Records with missing values in any of the selected fields were removed, and no imputation was applied, to avoid introducing artificial trends into the literature-based dataset.

**Table 3:** Variable definition and ranges in dataset.

Parameters (variable)	Mean	Std	Min	Median	Max
Fly Ash (kg/m^3^)	212.1	194.2	0.0	216.0	750.0
Meta-Kaolin (kg/m^3^)	67.5	145.0	0.0	0.0	411.0
Silica Fume (kg/m^3^)	3.9	28.0	0.0	0.0	428.0
Slag (kg/m^3^)	137.4	170.1	0.0	5.0	513.0
Gravel (kg/m^3^)	947.5	161.0	0.0	926.0	1311.0
Sand (kg/m^3^)	562.0	118.7	450.0	524.0	1200.0
NaOH (kg/m^3^)	51.4	18.8	31.0	48.0	114.0
Na_2_SiO_3_ (Liter/m^3^)	138.7	30.8	91.0	130.0	214.3
(Fibers - PP) (kg/m^3^)	0.3	0.9	0.0	0.0	6.1
(Fibers - SF) (kg/m^3^)	3.9	13.5	0.0	0.0	80.0
Curing age (Days)	20.3	9.8	7.0	28.0	48.0
Superplasticizer (Liter)	25.4	70.9	0.0	10.0	363.0
Water (Liter)	85.1	47.6	0.0	83.5	143.0
Compressive Strength (kg/cm^2^), (1 MPa = 10.1972 kg/cm²)	298.9	158.6	23.0	296.0	735.0

**Fig. 2:**
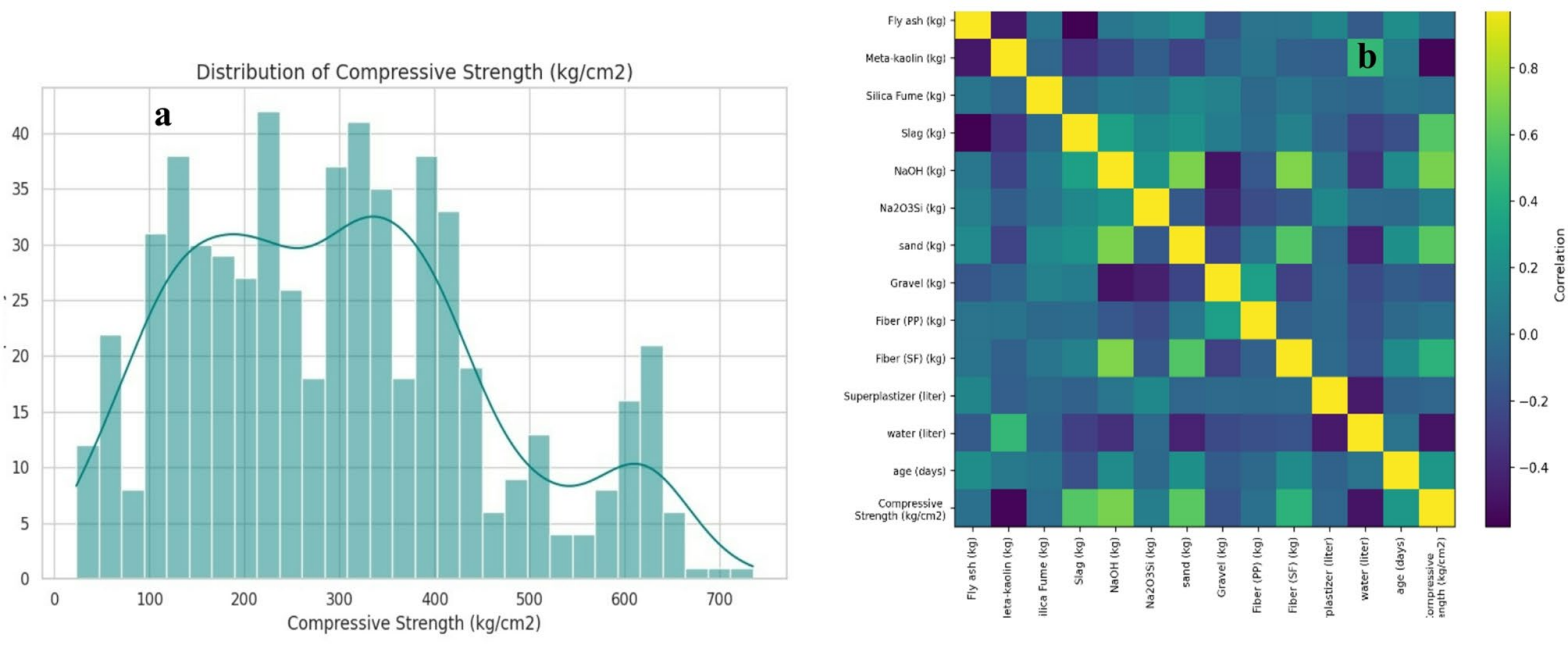
Dataset overview: compressive strength distribution and feature correlations.

Figure 2 summarizes the main statistical characteristics of the compiled dataset. Figure 2a shows the distribution of compressive strength values (kg/cm²). The strength results are spread over a wide range (approximately from low values up to about 700 + kg/cm²), indicating that the dataset includes mixes with markedly different performance levels. Most observations are concentrated in the mid-strength region, while fewer samples occur at very high strengths, which suggests an imbalanced but realistic distribution typical of literature-compiled datasets.

Figure 2b presents the Pearson correlation heatmap among the input variables and the compressive strength output. The diagonal line equals 1.0 because each variable is perfectly correlated with itself. Colors closer to yellow indicate stronger positive correlation (as one variable increases, the other tends to increase), while darker, blue tones indicate negative or weak correlation. Overall, the heatmap indicates that compressive strength is not controlled by a single factor; instead, it reflects combined effects of binder components, activator dosages, aggregates, water, and curing age. The presence of several moderate correlations between input variables also suggests interactions and co-variation (e.g., mix design trade-offs), supporting the need for nonlinear ML/DL models rather than relying only on simple linear relationships. The listed values present the specific gravity of the main materials used in the geopolymer concrete mixtures in Table 4. These properties support accurate mix proportioning and conversion between mass and volume during batching. The highest value corresponds to steel fiber, while water has the lowest, and the remaining binders and aggregates fall within typical ranges.

**Table 4:** Materials’ specific gravity.

Material	Value
Slag	2.9
Meta-kaolin	2.6
Fly ash	2.72
Silica fume	2.2
Water	1
Sand	2.64
Gravel	2.64
Fiber	7.8
Superplasticizer	1.05

## Machine learning models vs. deep learning models development

### Machine learning model architecture

ML enables models to learn patterns from data for prediction and is commonly categorized into supervised, unsupervised, and reinforcement learning, while recent progress has accelerated DL for modelling complex, high-dimensional relationships^33^. In this study, supervised ML was used to predict the compressive strength of geopolymer concrete (GPC) from mix proportions and material properties using models that capture nonlinear behavior, including Polynomial Regression, SVM (RBF), Decision Tree, Random Forest, and boosting ensembles (Gradient Boosting, XGBoost, LightGBM, and CatBoost), consistent with recent geopolymer/alkali-activated modeling studies^34^. All models were developed using a standard workflow of preprocessing, train test splitting, and evaluation with R², MSE, MAE, and MAPE^35^. In parallel, DL is increasingly applied in civil engineering for strength prediction and optimization from mixture and curing variables^36^, and it also supports defect detection (CNNs) and material discovery (GANs)^16^.

#### Deep learning model architecture

DL is increasingly important in civil engineering for improving material performance and supporting sustainability through data-driven analysis of complex datasets. By learning from variables such as mix proportions, curing duration, and ambient conditions, DL models can predict material behaviour and key properties like compressive strength, improving the reliability of Geopolymer design and optimization^36^. DL also strengthens quality control and infrastructure maintenance, where convolutional neural networks (CNNs) analyse high resolution images to detect and classify defects such as cracks and corrosion. This early defect identification enhances durability assessment and enables preventive maintenance before damage becomes severe^16^. Moreover, generative DL approaches (e.g., GANs) can propose new material formulations that satisfy target performance requirements, accelerating materials discovery while reducing cost and environmental impact compared with trial-and-error testing.

#### Artificial neural networks in deep learning

DL models were applied alongside traditional ML to capture the nonlinear relationships governing geopolymer concrete strength. Artificial neural networks (ANNs) are effective for complex multi-variable prediction in civil/materials engineering, including compressive strength estimation^37^. Two feed-forward ANN architectures were evaluated using ReLU activation: a Shallow ANN (64 − 32 neurons) and a Deep ANN (128-64-32 neurons), with Dropout (0.2) incorporated in the deep model to reduce overfitting and improve generalization. to bridge the gap between the interpretability of tree-based models and the representation learning capability of neural networks, the TabNet architecture was employed. TabNet is a deep learning model designed for tabular data that uses a sequential attention mechanism to select the most relevant features at each decision step, mimicking decision-tree feature selection while retaining end-to-end neural learning. Its inclusion enables a broader assessment of how modern attention-based deep learning compares with traditional ensemble methods in predicting geopolymer concrete compressive strength.

#### Comparative analysis of machine learning prediction models

**Fig. 3:**
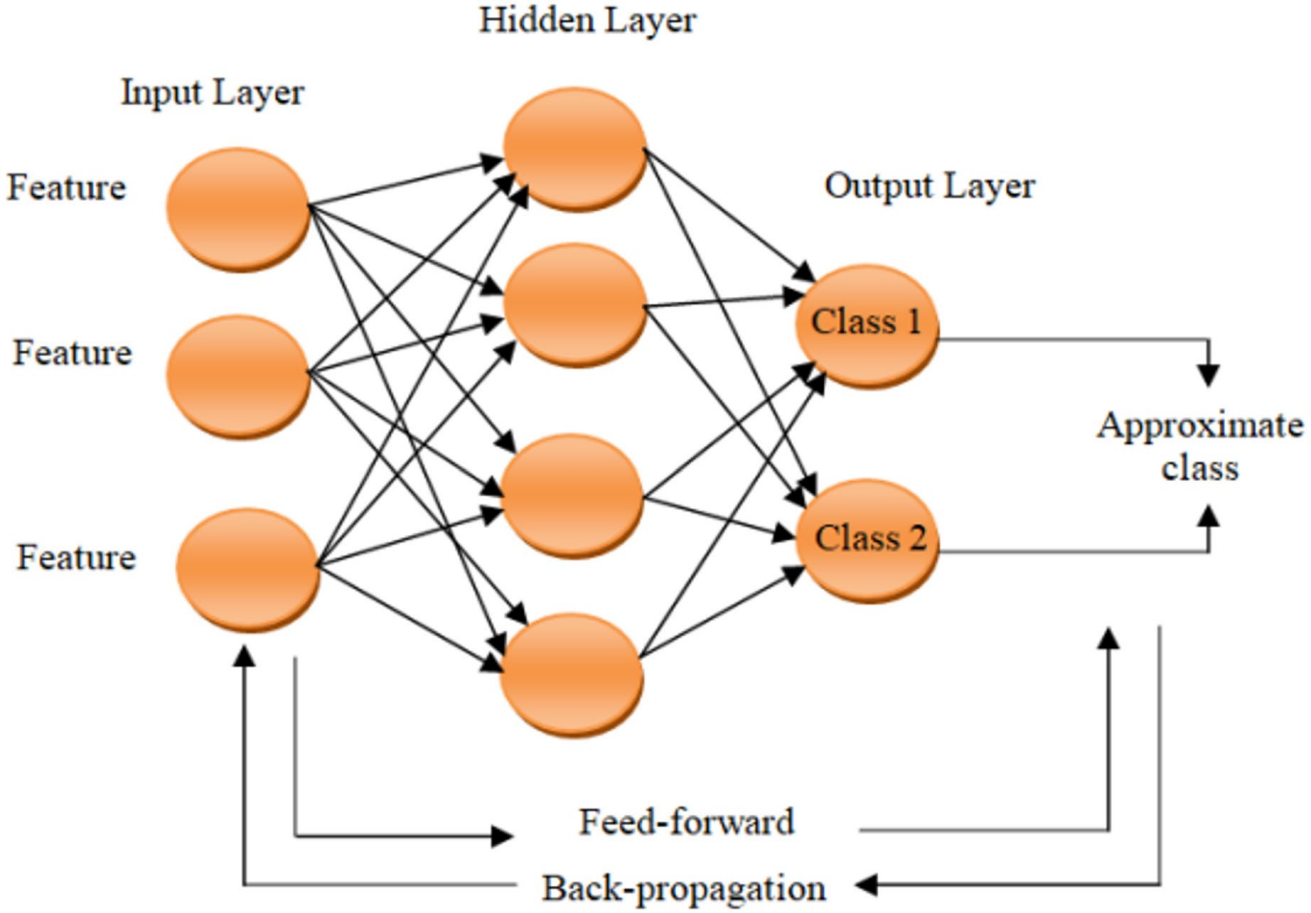
Conventional ANN architecture^38^.

A supervised learning framework was adopted to predict the compressive strength of geopolymer concrete from material and mix-design variables. The implemented models included Linear Regression, Polynomial Regression, Support Vector Machine, Random Forest, XGBoost, and Decision Tree, representing both linear and nonlinear learning approaches^33^. All models were developed using the same workflow, including preprocessing, feature preparation, 80/20 train–test splitting, and evaluation using R², MSE, MAE, and MAPE^35,34^. Figure 3 illustrates the adopted modeling architecture (conventional ANN architecture), while the comparative performance of the developed models is discussed in the Results and Discussion Sect.^38^.

### Data preprocessing

Data preprocessing plays a vital role in enhancing the accuracy and reliability of any models. In this study, the dataset was initially imported into a Pandas Data Frame to enable efficient manipulation and analysis. The dataset was then divided into features (independent variables) and the target variable (dependent output). To evaluate model performance, the data was split into training and testing subsets. This approach ensures a reliable assessment of the model on unseen data. The data obtained from the compressive strength tests were organized into a dataset for both training and testing randomly the ML models. The input features for the models included the weight percentages of fly ash and metakaolin in the binder, the volume percentage of steel fibers, the volume percentage of polypropylene fibers, and the molarity of the sodium hydroxide solution in the alkaline activator.

The database used in this study was compiled from multiple previously published experimental studies, which may inherently introduce heterogeneity due to differences in raw materials, curing conditions, specimen sizes, testing standards, and reporting practices. To improve consistency across the compiled dataset, a harmonization process was applied prior to model development. Only the most reported and consistently available input variables were retained, variable names and measurement units were standardized, and records with missing values in any of the selected input features or the target compressive-strength output were excluded from the final database. This harmonization and screening process reduced inconsistencies and enabled the construction of a unified dataset suitable for comparative ML and DL modeling. Nevertheless, some degree of inter study variability may remain and should be recognized as an inherent limitation of literature-based datasets.

After data cleaning and feature preparation, the input variables were normalized to ensure comparable feature scales and stable model training. The dataset was then divided into training and testing subsets using an 80/20 split with a fixed random state to ensure reproducibility.

### Programming tools

This study utilized Python as the primary programming language due to its powerful ecosystem of libraries for data analysis, visualization, and machine learning. All computational tasks were performed in Python used to develop and evaluate the machine learning and deep learning models in this study. The implementation relied on standard scientific computing libraries, including NumPy for numerical operations, Pandas for data handling and preprocessing, and Matplotlib for visualization and model-result plotting. All data processing, model training, and performance evaluation were performed within the Python environment. Machine learning models, such as Polynomial and Linear Regression, were built and evaluated using Scikit-learn, enabling efficient prediction of geopolymer compressive strength through a streamlined and effective workflow. Python’s extensive library ecosystem has significantly enhanced civil engineering research, particularly in data analysis and modelling. NumPy is essential for numerical computations in civil engineering, offering efficient array operations and matrix handling. It supports structural modeling and simulations such as stress analysis in concrete structures. Pandas enable fast, flexible data manipulation and are widely used for preprocessing structural engineering datasets. It improves model accuracy by handling missing values and transforming large datasets. Matplotlib is a core tool for visualizing civil engineering data, producing publication-ready plots. It helps illustrate trends in materials like geopolymer concrete under various conditions^39^.

### Machine learning, deep learning training

The compiled dataset was divided into training and testing subsets using an 80/20 split, (In the present study, model evaluation was conducted using a single 80/20 train–test split to ensure consistent comparison across all models. Although this approach enables direct benchmarking under identical conditions, it may remain sensitive to the specific partition used), with random_state = 42 to ensure reproducibility. For the ML models, the dataset was trained and evaluated using the implemented models of SVM, Decision Tree, Random Forest, XGBoost, and CatBoost. In the DL workflow, two feed-forward ANN architectures and one TabNet model were developed and assessed. Data preprocessing included standardization of the input features, while in the DL models the target variable was also standardized prior to training. The ANN models were trained using the Adam optimizer and mean squared error (MSE) loss function, with a maximum of 300 epochs, batch size of 32, validation split of 0.20, and early stopping with a patience of 20 epochs to prevent overfitting. For TabNet, the training process used the Adam optimizer with a learning rate of 0.02, StepLR scheduling (step_size = 10, gamma = 0.9), a maximum of 300 epochs, patience of 20, batch size of 256, and virtual batch size of 128. The final hyperparameters and training configurations adopted for all implemented ML and DL models are summarized in Table 5 to improve methodological transparency and reproducibility.

**Table 5:** Hyperparameters of the implemented ML and DL models.

Model	Key hyperparameters	Configuration method	Final value
SVM	Kernel, C, epsilon, feature scaling, target scaling	Predefined configuration	SVR with default RBF kernel, C = 10.0, epsilon = 0.1; input features scaled using StandardScaler; target scaled using TransformedTargetRegressor(StandardScaler())
Decision tree	criterion, splitter, max_depth, random_state	Default with user-defined seed	DecisionTreeRegressor(random_state = 42); all remaining parameters kept at library defaults
Random Forest	n_estimators, random_state	Partially user-defined configuration	RandomForestRegressor(n_estimators = 100, random_state = 42); all remaining parameters kept at library defaults
XGBoost	objective, random_state	Default with user-defined settings	XGBRegressor(objective = ‘reg: squarederror’, random_state = 42); all remaining parameters kept at library defaults
CatBoost	verbosity, random_state	Default with user-defined settings	CatBoostRegressor(verbose = 0, random_state = 42); all remaining parameters kept at library defaults
ANN_Shallow	Hidden layers, neurons, activation, optimizer, loss function, epochs, batch size, validation split, early stopping	Manually defined architecture	Dense layers: 64-ReLU, 32-ReLU, output layer 1-linear; optimizer = Adam; loss = MSE; epochs = 300; batch_size = 32; validation_split = 0.20; EarlyStopping(monitor = ‘val_loss’, patience = 20, restore_best_weights = True)
ANN_Deep	Hidden layers, neurons, dropout, activation, optimizer, loss function, epochs, batch size, validation split, early stopping	Manually defined architecture	Dense layers: 128-ReLU, Dropout(0.2), 64-ReLU, Dropout(0.2), 32-ReLU, output layer 1-linear; optimizer = Adam; loss = MSE; epochs = 300; batch_size = 32; validation_split = 0.20; EarlyStopping(monitor = ‘val_loss’, patience = 20, restore_best_weights = True)
TabNet	Optimizer, learning rate, scheduler, scheduler step size, scheduler gamma, mask type, max epochs, patience, batch size, virtual batch size	Manually defined architecture	TabNetRegressor(optimizer_fn = Adam, optimizer_params = {lr: 0.02}, scheduler_fn = StepLR, scheduler_params = {step_size: 10, gamma: 0.9}, mask_type = ‘entmax’, verbose = 0); max_epochs = 300; patience = 20; batch_size = 256; virtual_batch_size = 128; num_workers = 0; drop_last = False

### Performance evaluation

Key performance metrics for ML include Mean Absolute Error (MAE) represents the average absolute difference between predicted and actual values. Mean Squared Error (MSE) indicates the average of the squares of the errors, providing insight into the model’s prediction accuracy. R-squared (R²) measures the proportion of variance in the dependent variable that can be explained by the independent variables. R²- Adjusted is a modified version of the R² statistic that accounts for the number of predictors in a regression model. It provides a more accurate measure of how well the model explains the variability in the dependent variable. Mean Absolute Percentage Error (MAPE) is a metric used to evaluate the accuracy of a regression or forecasting model. It expresses prediction errors as a percentage, showing how far, on average, predicted values deviate from actual values. It is scale-independent and easy to interpret but can be sensitive to values close to zero. These metrics were used to assess the accuracy and reliability of the developed ML models in predicting the compressive strength of fiber-reinforced geopolymer concrete.

## Results and discussion

### Performance assessment of machine learning models

The compiled dataset results reveal the complex interplay between fiber reinforcement, alkaline activator molarity, and the compressive strength of geopolymer concrete. Similarly, the effect of polypropylene and steel fibers dosage on compressive strength at different molarities will be analyzed. The performance metrics obtained for the optimized ML models Table 6 indicate their capability in predicting the compressive strength of fiber-reinforced geopolymer concrete. The scatter plots visually confirm the strong correlation between the actual and predicted compressive strength values. The strengths and weaknesses of the developed ML models in capturing complex relationships within the dataset will be discussed, considering the influence of input features such as fiber type, dosage, and alkaline activator molarity.

**Table 6:** Machine learning performance results.

Models/comparison points	MAE	MSE	*R*^2^%	*R*^2^-Adjusted%	MAPE
SVM	20.300	914.221	0.962	0.957	0.137
Decision Tree	21.842	2112.046	0.912	0.901	0.082
Random Forest	19.758	1321.257	0.945	0.938	0.087
Light GBM	20.929	1248.084	0.948	0.942	0.117
Gradient Boosting	23.351	1366.247	0.943	0.936	0.154
XGBoost	16.516	986.472	0.959	0.954	0.072
CatBoost	14.740	759.484	0.968	0.964	0.076

Based on Table 6, the compared machine-learning models show clear differences in prediction accuracy and error levels. The CatBoost model achieves the strongest overall performance, producing the lowest MAE (14.740) and lowest MSE (759.484), while also delivering the highest R² (0.968) and adjusted R² (0.964), indicating both high accuracy and strong generalization. XGBoost ranks next, with competitive errors (MAE = 16.516, MSE = 986.472) and a high goodness of fit (R² = 0.959, adjusted R² = 0.954). The SVM model provides strong explanatory power (R² = 0.962), but with higher error levels (MAE = 20.300) and a relatively larger MAPE (0.137) compared to the best models. Random Forest and LightGBM show moderate performance, with R² values around 0.945–0.948 and MAE close to 19.758–20.929. In contrast, Decision Tree and Gradient Boosting yield the weakest results among this set, reflected by higher MAE (21.842 and 23.351, respectively) and lower R² (0.912 and 0.943). Overall, considering the combined criteria of minimum MAE/MSE and maximum R²/adjusted R², CatBoost is identified as the most reliable model for compressive strength prediction in this study.

### Actual vs. predicted strength analysis in ML models

Fig.s 4–6 compare the actual vs. predicted compressive strength obtained from the supervised ML models using the testing subset (20% of the dataset). In each sub-Fig., the measured strength values are plotted on the horizontal axis and the corresponding model predictions on the vertical axis. The red 45° dashed line represents the ideal agreement line; therefore, the closeness of the points to this line provides a direct visual indication of prediction accuracy.

**Fig. 4:**
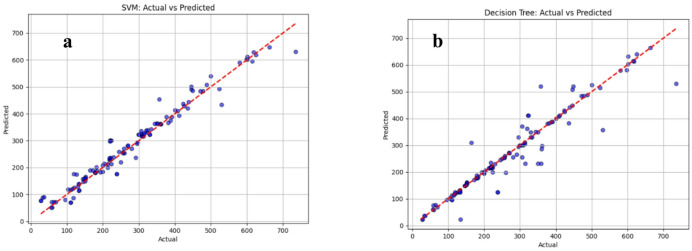
Actual Vs predicted compressive strength values (**a**) support vector machines (SVM) model, (**b**) decision tree model.

**Fig. 5:**
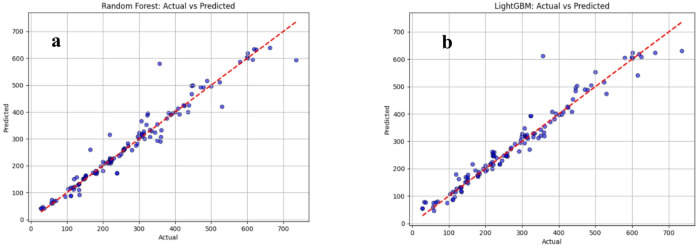
Actual Vs predicted compressive strength values (**a**) random forest model, (**b**) light GBM model.

The scatter patterns show noticeable differences among the models. In Fig. 4a (SVM), most points cluster close to the ideal line across a wide strength range, indicating strong predictive capability, with some dispersion appearing mainly at higher-strength samples. In Fig. 3b (Decision Tree), the spread around the diagonal is more pronounced and several points deviate substantially, reflecting weaker generalization and higher sensitivity to localized splits in the feature space. Figure 5a (Random Forest) demonstrates improved stability compared to the single Decision Tree, as the ensemble averaging reduces extreme deviations; however, some under- and over-predictions remain, particularly in the mid-to-high strength region. Figure 5b (LightGBM) shows a similar trend but with slightly tighter clustering than Random Forest in most regions, consistent with the advantages of boosting-based learning in capturing nonlinear relationships.

**Fig. 6:**
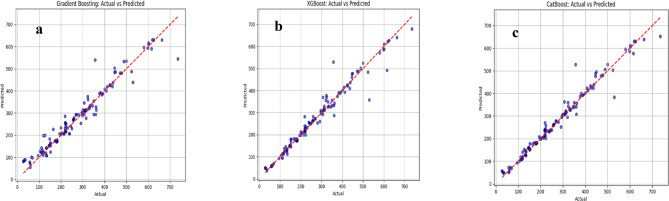
Actual Vs predicted compressive strength values (**a**) gradient boosting model, (**b**) XGBoost model, (**c**) CatBoost.

Figure 6 highlights the boosting-based family more clearly. Gradient Boosting Fig. 6a presents moderate agreement with the reference line but still exhibits a wider scatter, indicating comparatively higher prediction errors. In contrast, XGBoost, Fig. 6b produces a noticeably tighter distribution around the diagonal, suggesting stronger fitting and better control of bias–variance trade-off. Finally, CatBoost, Fig. 6c shows the closest alignment to the ideal line with reduced dispersion across most of the dataset, indicating the most consistent prediction behavior among the evaluated models.

These visual observations align with the statistical outcomes reported in Table 5 boosting-based ensembles especially CatBoost and XGBoost, demonstrate the strongest predictive performance (lower error and higher goodness-of-fit), while single-tree and some baseline learners show larger scatter and reduced accuracy. Overall, the Fig.s confirm that ensemble and boosting approaches provide the most reliable compressive strength predictions for the studied geopolymer concrete dataset.

### SHAP-based feature importance of machine learning

**Fig. 7:**
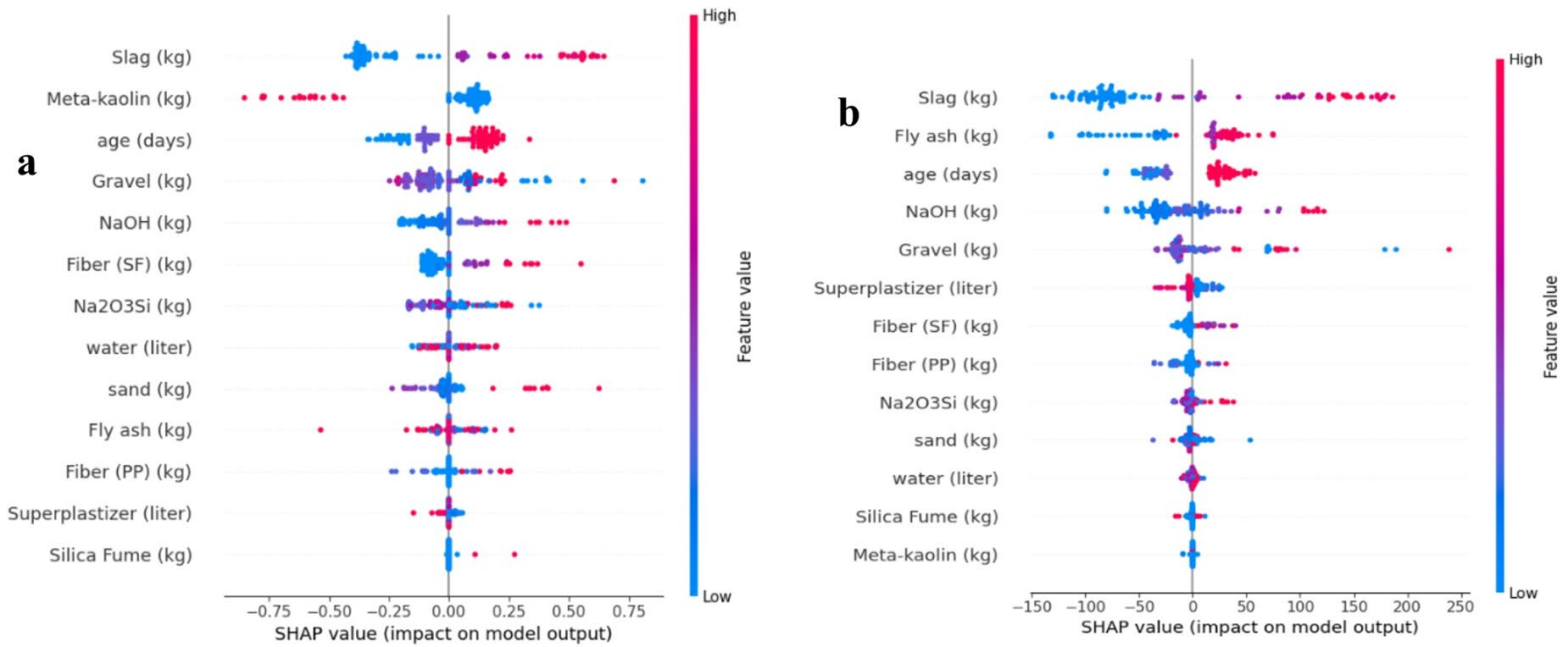
Feature importance analysis using SHAP values with (**a**) support vector machines (SVM) model, (**b**) decision tree model.

**Fig. 8:**
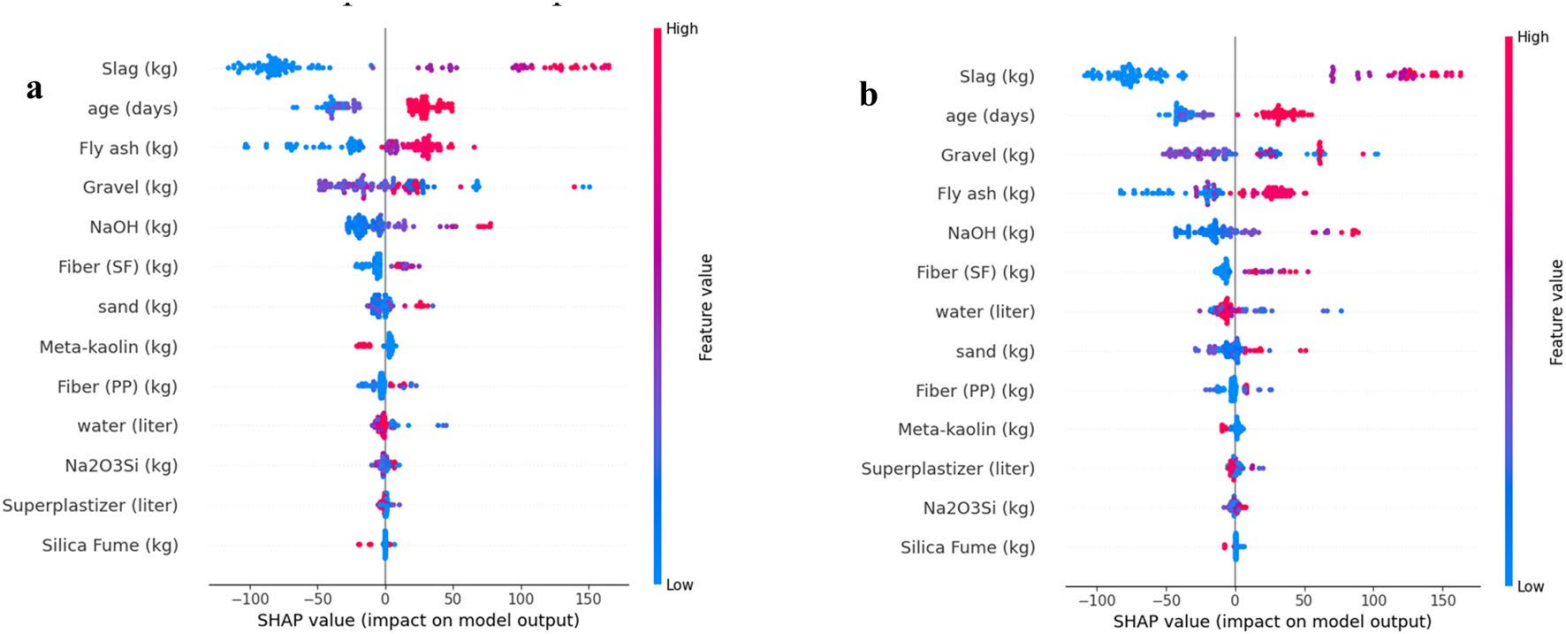
Feature importance analysis using SHAP values with (**a**) random forest model, (**b**) light GBM model.

**Fig. 9:**
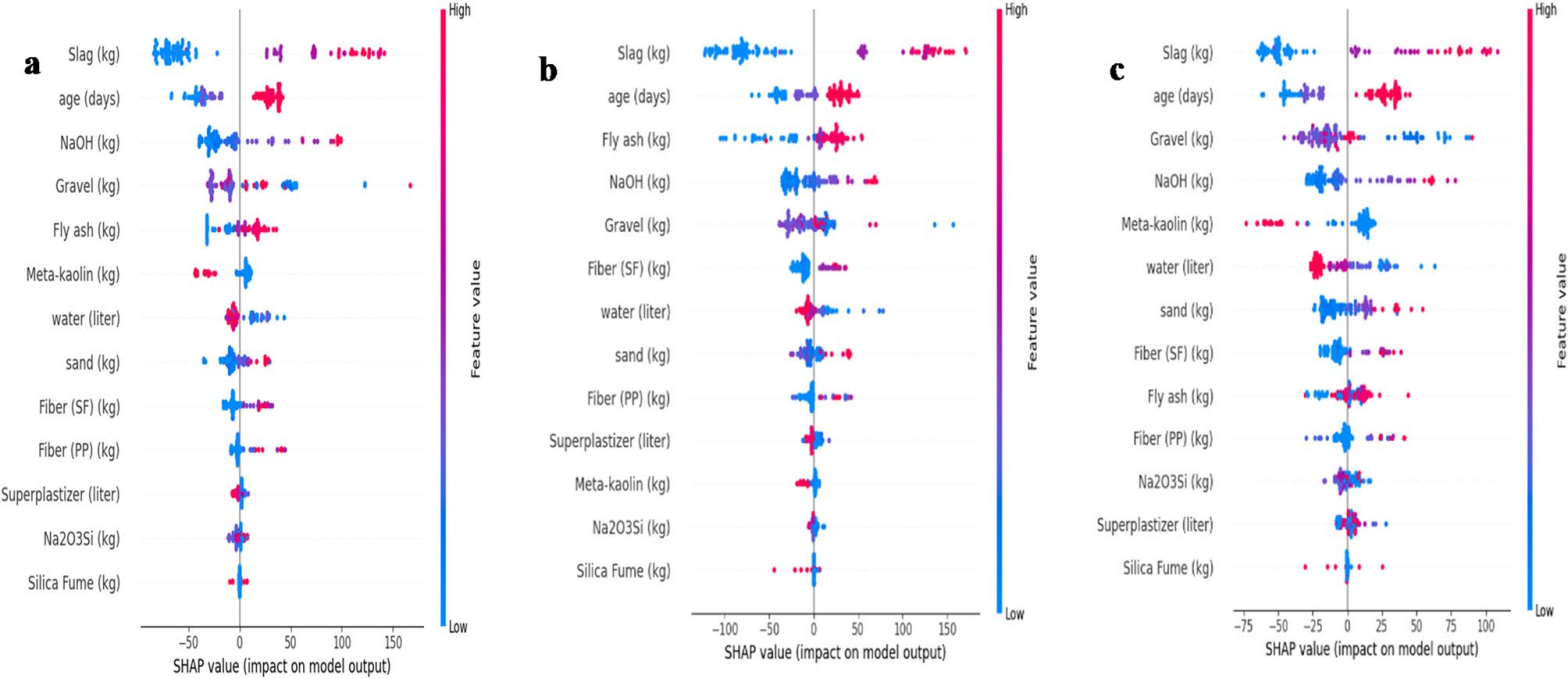
Feature importance analysis using SHAP values with (**a**) gradient boosting model, (**b**) XGBoost model, (**c**) CatBoost.

Figures. 7, 8 and 9 present SHAP (SHapley Additive exPlanations) summary plots for the evaluated ML models (SVM, Decision Tree, Random Forest, LightGBM, Gradient Boosting, XGBoost, and CatBoost). In each plot, features are ranked from top to bottom by their overall influence on the predicted compressive strength. The horizontal axis represents the SHAP value (magnitude and direction of impact on the model output), while the color scale reflects the feature value (blue = low, red = high).

Across all models, slag content consistently appears as the most influential parameter, from a material-science perspective, the dominance of slag content and curing age in the SHAP analysis is consistent with the known mechanisms governing geopolymer concrete strength development. Slag is rich in calcium and therefore plays a major role in accelerating reaction kinetics and promoting the formation of calcium–alumino–silicate–hydrate (C–A–S–H)-type gels in addition to geopolymeric binding products. This contributes to faster matrix densification, reduced porosity, and improved early-age strength. Curing age is similarly critical because compressive strength in geopolymer systems develops progressively with the advancement of geopolymerization, continued gel formation, and refinement of the internal pore structure over time. Accordingly, the high SHAP importance assigned to slag and age reflects not only their statistical influence in the dataset, but also their fundamental role in controlling the microstructural evolution and load-bearing capacity of geopolymer concrete. showing the widest SHAP spread and the strongest contribution to prediction variability. In most cases, higher slag values (red points) are concentrated on the positive SHAP side, indicating that increasing slag content generally drives the predicted compressive strength upward. The curing age is also repeatedly ranked among the top predictors (often second), where higher ages tend to contribute positively to strength prediction, reflecting the role of ongoing reaction development and matrix densification with time. After slag and age, the models highlight a secondary group of influential variables, most notably gravel content, NaOH content, and the precursor-related parameters (fly ash and meta-kaolin). The ranking among these variables varies slightly by algorithm for example, Decision Tree assigns relatively higher importance to fly ash, while CatBoost increases the relative contribution of gravel and NaOH, but the same group remains consistently relevant across the ensemble/boosting methods. This stability supports the physical expectation that compressive strength is strongly governed by a combination of binder chemistry, activator dosage, and aggregate skeleton. Overall, the SHAP-based interpretation for the deep learning models confirms that compressive strength is governed mainly by precursor composition and curing/activation conditions, whereas fibers and minor constituents play a less dominant role in the predicted output.

the SHAP analyses in Figs. 7, 8 and 9 confirm that the prediction of geopolymer concrete compressive strength is primarily governed by slag content and curing age, followed by aggregate/activator/binder proportions, while fibers and minor constituents provide secondary refinements to model output.

### Performance assessment of ANN models

Table 7 presents the prediction accuracy of the developed DL models (ANNs and TabNet) in terms of MAE, MSE, R^2^, adjusted R^2^, and MAPE. Overall, the TabNet model provides the best performance among the evaluated deep learning approaches, achieving the lowest prediction error and the strongest goodness-of-fit. Specifically, TabNet records MAE = 18.27, MSE = 765.80, R^2^ = 0.968, and adjusted R^2^ = 0.960, indicating superior prediction capability and excellent generalization. In contrast, the ANN configurations show higher error rates and reduced accuracy. The ANN Shallow model serves as an intermediate performer with an MAE of 22.24 and an R^2^ of 0.937. The ANN Deep model demonstrates the weakest performance among the three, recording the highest errors (MAE = 30.16, MSE = 2146.17) and the lowest explanatory power (R^2^ = 0.911). This hierarchy is also evident in the relative error values, where TabNet achieves the lowest MAPE of 0.101, compared with 0.120 for ANN Shallow and 0.203 for ANN Deep. These results indicate that for the current dataset, the attention-based feature selection of TabNet is more effective than standard neural architectures, and that increasing network complexity through a deeper ANN leads to a decline in prediction accuracy.

**Table 7:** ANN performance results.

Models/comparison points	MAE	MSE	*R* ^2^	*R*^2^-Adjusted	MAPE
ANN_Shallow	22.24	1501.915	0.937	0.930	0.120
ANN_Deep	30.15	2146.165	0.911	0.900	0.203
TabNet	18.27	765.800	0.968	0.960	0.101

### Deep learning model evaluation and comparison

**Fig. 10:**
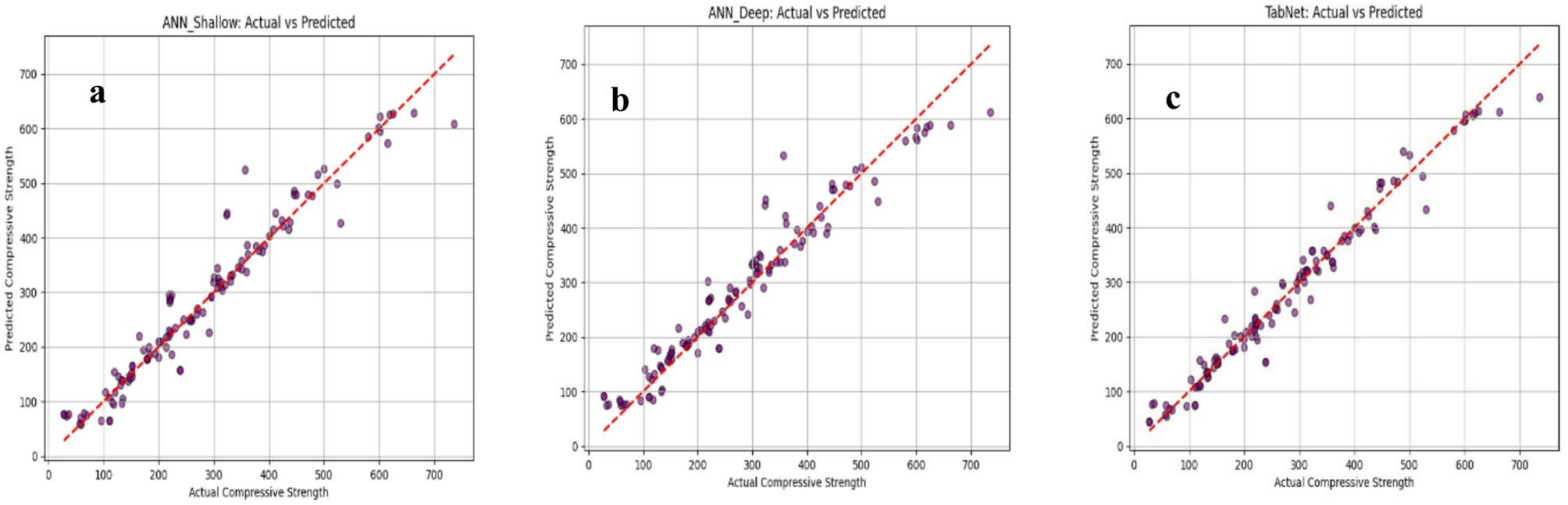
Actual Vs predicted compressive strength values (**a**) ANN_Shallow model, (**b**) ANN_Deep model, (**c**) TabNet model.

Figure 10 compares the actual versus predicted compressive strength results for the three deep learning models: (a) ANN_Shallow, (b) ANN_Deep, and (c) TabNet. While all models demonstrate a positive relationship with most points distributed around the 45° reference line, the TabNet model (Fig. 10c) provides the most precise fit. It achieves the highest coefficient of determination (R^2^) of 0.968 and the lowest Mean Absolute Error (MAE) of 18.27. This superior performance is visually confirmed by the tightest clustering of data points around the ideal line across the entire strength range. In contrast, the ANN_Deep model (Fig. 10b) exhibits the greatest dispersion and more noticeable departures from the reference line, particularly at higher strength values, reflecting its higher MAE of 30.15 and lower R^2^ of 0.911. The ANN_Shallow model (Fig. 10a) represents an intermediate performance, achieving an R^2^ of 0.937 and an MAE of 22.24. These results, summarized in the accompanying data, confirm that TabNet offers the highest prediction consistency and lowest overall error among the evaluated architectures.

The comparatively weaker performance of ANN_Deep can be partly attributed to the limited size of the available dataset relative to the complexity of the architecture. Deep learning models, particularly deeper neural networks, generally benefit from larger and more diverse datasets to learn stable nonlinear patterns effectively. For moderate-sized tabular datasets such as the present one, simpler neural architectures or boosting-based ML models may therefore offer better generalization than deeper networks.

The inferior performance of ANN_Deep can be attributed to the mismatch between model complexity and the available dataset scale. With a dataset of 594 literature-derived samples and a heterogeneous tabular feature space, the deeper architecture likely introduced excessive parameterization relative to the available information content. As a result, optimization became less stable and convergence less efficient than in the shallow ANN. For this dataset, the simpler network was sufficient to capture the dominant nonlinear relationships, whereas additional depth increased training difficulty without improving generalization. This interpretation is consistent with the larger prediction dispersion and less stable loss evolution observed for ANN_Deep.

**Fig. 11:**
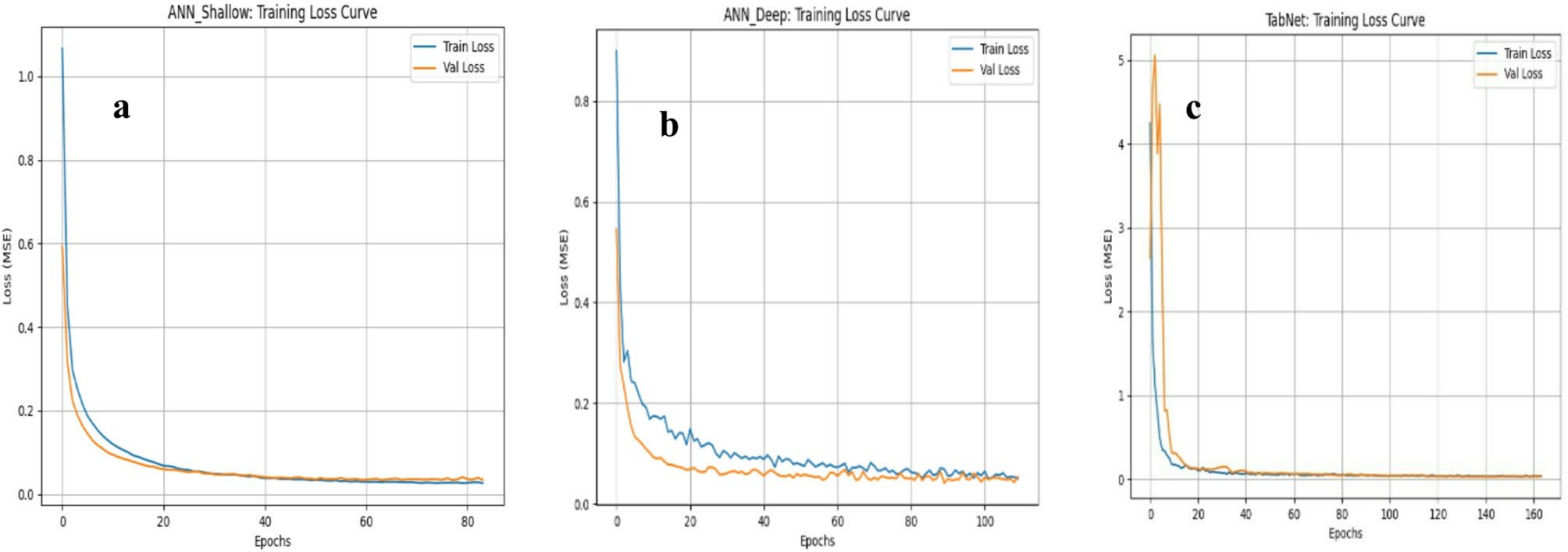
Training loss curve values of (**a**) ANN_Shallow model, (**b**) ANN_Deep model, (**c**) TabNet.

The training and validation loss curves Fig. 11 further support this comparison. For the ANN Shallow model Fig. 11a, both training and validation losses decrease rapidly during the early epoch and then stabilize smoothly at low values, demonstrating stable learning and good generalization behavior. In contrast, the ANN Deep model Fig. 11b shows a slower reduction in loss and more noticeable fluctuations, with a persistent gap between training and validation curves in the earlier stages, suggesting a higher sensitivity to model complexity and a greater tendency toward less stable convergence.

The loss curves in Fig. 11 further support the numerical comparisons reported in Table 6. For ANN Shallow (Fig. 11a), both training and validation losses decrease rapidly during the early epochs and then converge smoothly to a low, stable plateau with only a small gap between the two curves, indicating stable learning and good generalization. In ANN Deep (Fig. 11b), the losses also decline, but the convergence is slower and the training curve remains consistently higher than the validation curve over a longer training period, reflecting less stable optimization and reduced predictive consistency compared with the shallow network. For TabNet (Fig. 11c), the loss starts at a much higher level and drops sharply in the initial epochs, yet it stabilizes at a comparatively higher plateau than the ANN models, suggesting weaker overall fit to the dataset. Overall, the convergence behavior confirms that the shallow ANN reaches a lower and more stable validation loss, whereas increasing model complexity (deeper ANN or TabNet) does not yield improved learning or generalization for the current dataset.

### SHAP-based feature importance of deep learning models

**Fig. 12:**
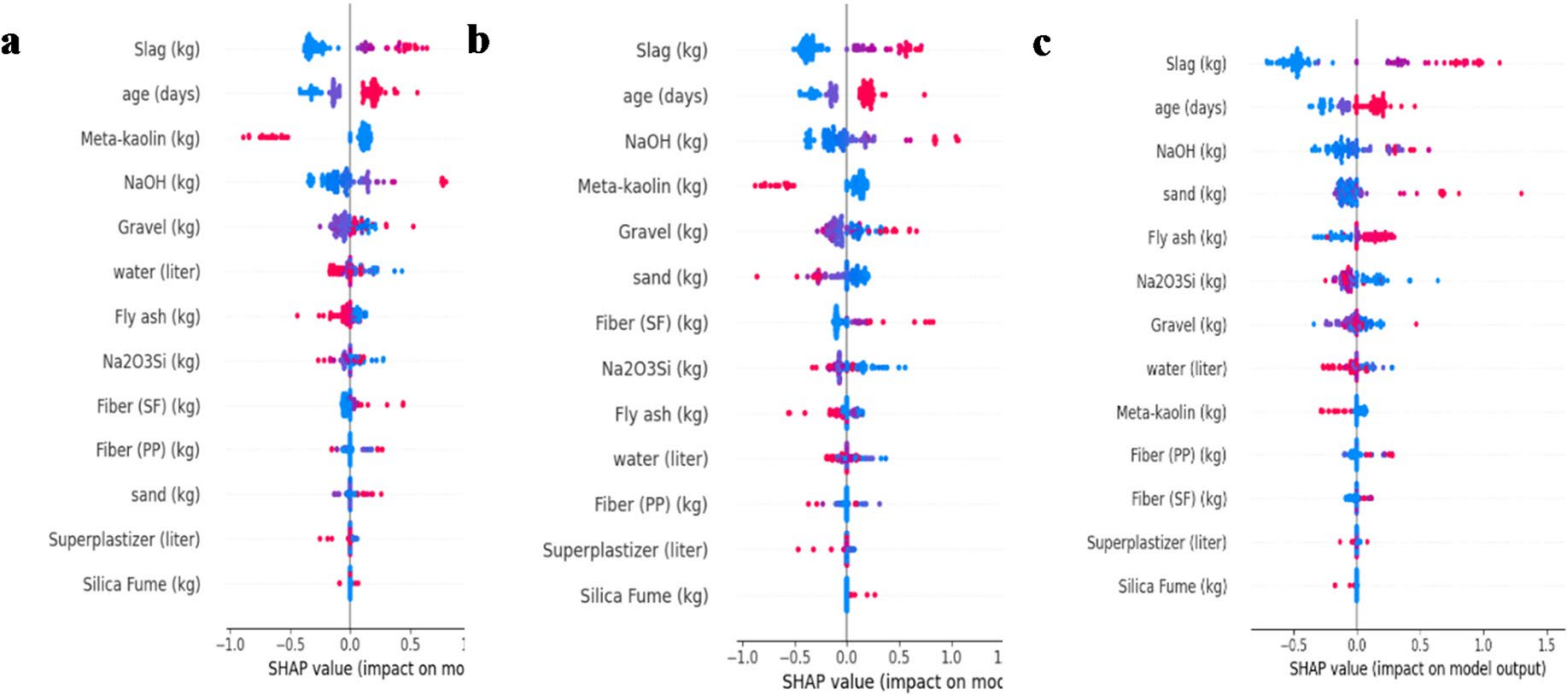
Feature importance analysis using SHAP Values with (**a**) ANN_Shallow Model, (**b**) ANN_Deep Model, (**c**) TabNet Model.

Figure 12 presents the SHAP summary plots for the deep learning models, illustrating the relative contribution of each input variable to compressive strength prediction for (a) ANN Shallow, (b) ANN Deep, and (c) TabNet. Across all sub-Figures, slag is consistently ranked as the most influential feature, showing the widest SHAP value spread and therefore the strongest impact on model output. The next most important parameters are largely associated with geopolymer reaction development and activator/binder chemistry most notably curing age, NaOH, and meta-kaolin which repeatedly appear among the top ranked predictors in both ANN architectures and TabNet. Variables such as gravel, water, and Na₂SiO₃ exhibit moderate SHAP influence, indicating secondary effects related to aggregate skeleton characteristics and the overall liquid/activator balance in the mixture. In contrast, steel fiber and polypropylene fiber generally show smaller SHAP ranges and lower rankings, suggesting a comparatively limited contribution to compressive strength prediction within this compiled dataset.

The SHAP analysis further showed that the fiber-related variables, namely steel fibers and polypropylene fibers, had a moderate but clearly lower influence on compressive strength compared with slag content, curing age, and NaOH concentration. From a civil engineering perspective, this result is reasonable because compressive strength in geopolymer concrete is governed mainly by matrix formation, binder chemistry, activator concentration, and curing conditions, all of which directly affect gel development, porosity, and matrix densification. By contrast, discrete fibers contribute primarily through crack-bridging and post-cracking restraint mechanisms, which are more effectively mobilized under tensile and flexural loading than under uniaxial compression. Therefore, although SF and PP fibers may provide limited enhancement in compressive strength by delaying crack propagation and restricting microcrack growth, their contribution remains secondary relative to the binder–activator–curing system. This mechanical interpretation is consistent with the SHAP rankings obtained in the present study.

The TabNet SHAP plot (Fig. 12c) follows the same overall pattern observed for the ANN models emphasizing binder/activator and curing parameters as primary drivers, while also showing strong sensitivity to slag and noticeable contributions from aggregate related variables. Overall, the SHAP-based interpretation for the deep learning models confirms that compressive strength is governed mainly by precursor composition and curing/activation conditions, whereas fibers and minor constituents play a less dominant role in the predicted output.

### Comparison between machine learning and deep learning models

The developed machine learning (ML) and deep learning (DL) models were evaluated using the same dataset split and performance metrics. Overall, the ML models achieved higher predictive accuracy than the DL models in the present study. Among the ML approaches, CatBoost delivered the best overall performance, as evidenced by the lowest error values and the highest coefficient of determination, confirming its strong generalization capability on the testing dataset. Within the DL group, TabNet achieved the best predictive performance, outperforming both ANN_Shallow and ANN_Deep in terms of overall accuracy. Although ANN_Shallow showed more stable convergence behavior than ANN_Deep, its final predictive accuracy remained lower than that of TabNet. The weaker performance of ANN_Deep further indicates that increasing network depth did not improve generalization for the current dataset. Collectively, these findings demonstrate that boosting-based ML models provide the most reliable framework for compressive-strength prediction in this dataset, while among the DL models, TabNet emerged as the strongest benchmark, showing that attention-based tabular learning can be effective even though it did not surpass the best ML approach.

In this context, the present results suggest that the advantage of deep learning is not automatic, but depends strongly on data volume, feature richness, and the match between model complexity and dataset structure.

In summary, CatBoost and XGBoost emerged as the two best-performing ML models, with CatBoost ranking first (MAE = 14.740, MSE = 759.484, R² = 0.968, adjusted R² = 0.964) and XGBoost ranking second (MAE = 16.516, MSE = 986.472, R² = 0.959, adjusted R² = 0.954). Among the DL models, TabNet was the strongest performer (MAE = 18.27, MSE = 765.80, R² = 0.968, adjusted R² = 0.960), followed by ANN_Shallow (MAE = 22.24, R² = 0.937), whereas ANN_Deep showed the weakest generalization. Overall, the ML group remained superior, indicating that boosting-based methods were better suited to the present moderate-sized tabular dataset than the tested ANN architectures.

### Limitations and future work

Despite the promising predictive performance achieved in this study, several limitations should be acknowledged. The dataset was compiled from previously published studies and therefore reflects inter-study variability in materials, testing conditions, and reporting practices. In addition, although the dataset size was sufficient for comparative modeling, it remains moderate for more complex deep-learning applications. The study was also limited to compressive-strength prediction and did not include external validation using newly generated experimental data. Future research should focus on constructing larger and more standardized geopolymer concrete databases, validating the developed models against independent experimental datasets, and exploring hybrid, physics-informed, and uncertainty-aware modeling frameworks. Extending the proposed approach to other mechanical and durability properties would also improve its practical relevance.

## Conclusion

- A consolidated literature-based dataset of 594 geopolymer concrete mix designs was established, incorporating the most consistently reported mix parameters and curing age to support data-driven compressive strength prediction.
- The comparative ML evaluation showed clear performance differences; CatBoost provided the most accurate and generalizable predictions among ML models (MAE = 14.740, MSE = 759.484, R² = 0.968, adjusted R² = 0.964), followed by XGBoost with competitive accuracy.
- Deep learning benchmarks confirmed that these models effectively capture the nonlinear trends in the dataset, with predictions closely aligned with the 45° reference line. However, the results indicate that increasing standard network depth was not advantageous for this specific dataset, as the ANN Shallow model achieved better predictive accuracy than the more complex ANN Deep configuration.
- TabNet, utilizing an attention-based feature selection mechanism specifically designed for tabular learning, produced the superior fit among all deep learning models, recording the lowest prediction error (MAE = 18.27, MSE = 765.80) and the highest explanatory power (R^2^ = 0.968).
- Visual comparisons of actual vs. predicted values were consistent with the numerical metrics: TabNet exhibited the tightest clustering around the 45° line, while ANN Shallow and ANN Deep showed increasing dispersion and reduced consistency at higher strength ranges.
- SHAP-based interpretation demonstrated stable governing factors across models: compressive strength prediction is primarily controlled by slag content and curing age, followed by binder/activator/aggregate proportions, whereas fiber contents and minor constituents mainly provide secondary adjustments to the predicted output.
- The proposed framework (training/testing evaluation + model ranking + SHAP interpretation) offers a practical pathway to reduce trial-and-error in geopolymer mixture design and to support more efficient strength estimation and mix optimization.
- The performance of the developed models was evaluated using several statistical metrics and actual-versus-predicted comparisons; however, the study did not include detailed residual-error distribution analysis, prediction interval estimation, or formal uncertainty quantification. Thus, the reported results represent a comparative measure of predictive accuracy rather than a full characterization of uncertainty for practical engineering deployment.
- A literature-based database containing 594 geopolymer concrete mixes was compiled and harmonized from published studies to support comparative predictive modeling.
- Among the evaluated machine-learning models, CatBoost achieved the best overall predictive performance for compressive-strength estimation.
- Within the deep-learning models, TabNet showed the strongest performance, whereas deeper ANN architecture did not provide superior accuracy for the available dataset size.
- The results indicate that boosting-based ML models were more robust than the tested DL models for this moderate-sized heterogeneous tabular dataset.
- SHAP analysis showed that slag content, curing age, and activator-related parameters were the most influential variables governing compressive-strength prediction across the developed models.
- The proposed framework can support preliminary strength estimation and comparative mix screening, although further validation using larger and more standardized datasets is still needed.

## Supplementary Information

Supplementary Material 1

## Data Availability

All data generated or analyzed during this study are included in this published article.

## Code availability

The custom code used for the data analysis and to generate the results presented in this study is provided as part of the Supplementary Information files accompanying this manuscript.
