# Supplementary material for: Comparative evaluation of machine learning and deep learning approaches for compressive strength prediction of geopolymer concrete
Source: Sci Rep. 2026 May 7;16:14652. doi: 10.1038/s41598-026-50705-w (PMC13153404; doi:10.1038/s41598-026-50705-w)
Supplement: Supplementary file 1 — Supplementary Material 1 [file 41598_2026_50705_MOESM1_ESM.zip › ML/README.pdf]

README: Machine Learning for Geopolymer Analysis

This folder contains the custom Python code used for the data analysis, model training, and interpretability (SHAP) results presented in the manuscript.

1. Overview

The script ML2.py performs a comparative study of eight machine learning models to predict the compressive strength of geopolymers. It handles:

- **Data Preprocessing:** Automated cleaning and train-test splitting (80/20).
- **Model Training:** Implements SVM, Polynomial Regression, Decision Tree, Random Forest, LightGBM, Gradient Boosting, XGBoost, and CatBoost.
- **Evaluation:** Calculates MAE, MSE, R^2, Adjusted R^2, and MAPE.
- **Visualizations:** Generates "Actual vs. Predicted" plots for every model.
- **Interpretability:** Generates SHAP (SHapley Additive exPlanations) summary plots to explain feature importance.

2. Requirements

To run this script, you need Python 3.x and the following libraries:

pip install pandas numpy matplotlib scikit-learn xgboost lightgbm catboost shap openpyxl

3. Usage Instructions

1. **Data Preparation:** Ensure your dataset is in an Excel format (named Data_test.xlsx) with the features in the initial columns and the target variable (Compressive Strength) in the final column.
2. **Path Configuration:** * Open ML2.py.
    - Modify the work_dir variable (Line 31) to match the folder path on your local machine where the data is stored.
3. **Execution:** Run the script using:

python ML2.py

4. Output

The script will automatically create two sub-folders and an Excel file in your working directory:

- **/Model_Plots:** Contains regression plots (Actual vs. Predicted) for all models.
- **/SHAP_Plots:** Contains SHAP summary plots illustrating the global impact of each feature.

- **Model_Performance_Metrics_v3.xlsx:** A summary table of all statistical error metrics for the evaluated models.

5. Contact

For any technical queries regarding the implementation of this code, please contact the corresponding author, **Hesham Ezz**.
