# Supplementary material for: Comparative evaluation of machine learning and deep learning approaches for compressive strength prediction of geopolymer concrete
Source: Sci Rep. 2026 May 7;16:14652. doi: 10.1038/s41598-026-50705-w (PMC13153404; doi:10.1038/s41598-026-50705-w)
Supplement: Supplementary file 1 — Supplementary Material 1 [file 41598_2026_50705_MOESM1_ESM.zip › ANN/README.pdf]

README: Deep Learning & TabNet for Geopolymer Analysis

This folder contains the custom Python code used for the deep learning analysis and feature importance evaluations presented in the manuscript.

1. Overview

The script ANN3.py implements advanced neural network architectures to predict the compressive strength of geopolymers. It compares three distinct approaches:

- **Shallow ANN:** A feed-forward neural network with two hidden layers (64 and 32 neurons).
- **Deep ANN:** A more complex architecture with three hidden layers ($128, 64, 32$ neurons) and **Dropout regularization** to prevent overfitting.
- **TabNet:** A deep tabular data architecture that uses sequential attention to mimic the decision-making process of tree-based models while maintaining neural network benefits.

2. Requirements

This script requires **TensorFlow** (for ANNs) and **PyTorch** (for TabNet). You can install all dependencies via pip:

pip install pandas numpy matplotlib scikit-learn tensorflow pytorch-tabnet torch shap openpyxl

3. Usage Instructions

1. **Data Preparation:** The script expects an Excel file named Data_test.xlsx.
    - **Features:** All columns except the last one.
    - **Target:** The final column (Compressive Strength).
2. **Path Configuration:** * Open ANN3.py.
    - Update the work_dir variable (Line 24) to point to the directory containing your data file.
3. **Execution:** Run the script using:

python ANN3.py

4. Key Features in the Code

- **Scaling:** Both features (X) and targets (y) are standardized using StandardScaler for stable neural network convergence. Results are automatically inverse-transformed back to original units for error calculation.

- **Early Stopping:** Keras models use EarlyStopping to monitor validation loss and prevent overfitting.
- **Loss Curves:** The script generates training vs. validation loss plots to verify model health and convergence.
- **Interpretability:** Uses shap.KernelExplainer to calculate SHapley Additive exPlanations for all deep learning models.

5. Output

Results are organized into the following generated files:

- **/DL_Model_Plots:** * Actual_vs_Predicted.png: Scatter plots for each model.
    - Training_Loss.png: Convergence curves for each model.
- **/DL_SHAP_Plots:** Global feature importance summary plots.
- **DL_Model_Performance_Metrics.xlsx:** Comprehensive table including MAE, MSE, R^2, Adjusted R^2, and MAPE.

6. Contact

For any technical queries regarding the implementation of this code, please contact the corresponding author, **Hesham Ezz**.
